# Understanding *understanding* in psychiatry

**DOI:** 10.1177/0957154X231163275

**Published:** 2023-04-24

**Authors:** Joseph Gough

**Affiliations:** University of Durham, UK

**Keywords:** Explanation, mind–body problem, phenomenology, psychiatry, understanding

## Abstract

Originally put forward to defend history from the encroachment of physics, the distinction between understanding and explanation was built into the foundations of Karl Jaspers’ ‘phenomenological’ psychiatry, and it is revised, used and defended by many still working in that tradition. On the face of it, this is rather curious. I examine what this notion of ‘understanding’ amounts to, why it entered and remains influential in psychiatry, and what insights for contemporary psychiatry are buried in the notion. I argue that it is unhelpfully associated with the view that the mental is epistemologically and methodologically autonomous, but that it nevertheless highlights an important lacuna in many views of psychiatry and the scientific study of humans more generally.

## Introduction

Phenomenological approaches to psychiatry are undergoing something of a renaissance, and in my view this is good news for psychiatry. However, my optimism is tempered by the enthusiasm of the tradition for certain aspects of Karl Jaspers’ framework that are unmotivated, unhelpful, and incompatible with Husserlian conceptions of phenomenology.

Jaspers argued for a ‘phenomenological’ approach to psychiatry, a label that he adopted to acknowledge his debt to Husserl. Essential to the foundations of Jasperian phenomenological psychiatry is a distinction between explanation (*Erklären*) and understanding (*Verstehen*). Jasper’s version of this distinction owes much to the antipositivist tradition of Dilthey, Rickert and Weber. In that tradition, the distinction is used to explicate the difference in methods and goals between the human sciences (*Geisteswissenschaften*), associated with understanding, and the natural sciences (*Naturwissenschaften*), associated with explanation.

It is far from clear why this distinction should play any part in psychiatry of any form. I will argue that Jaspers’ motivation for adopting the distinction cannot plausibly be accounted for using the justifications on offer in the antipositivist tradition. Instead, I will argue, Jaspers appears to see the distinction as a way of circumventing a perceived mind–body problem. I will further suggest that best practice in psychiatry counts against both the distinction and Jaspers’ motivation for using it, even according to recent reconstructions of best practice by those friendly to phenomenological approaches and those attempting to save the distinction.

However, there is a risk of losing both baby and bathwater. Jaspers uses the notion of understanding in two main ways. One is to set up a distinction among disorders and symptoms that he uses to limit what he perceives as the encroachment of biological approaches on the domain of the mental. This is not a good idea, and nor are recent attempts to reconstrue this distinction as a matter of practitioner attitudes or classes of methods. The other, however, is as the label for a proposed scientific method built upon ordinary empathy. Whether or not Jaspers succeeded in his specific proposal, there is great merit in the core idea of offering methodological guidance for making rigorous and reliable use of ordinary empathy to learn things about people with psychiatric conditions.

## What is understanding?

Jaspers introduces understanding in contrast to explanation. In understanding ‘[w]e sink ourselves into the psychic situation and understand genetically by empathy how one psychic event emerges from another’; in explanation, ‘[w]e find by repeated experience that a number of phenomena are regularly linked together, and on this basis we explain causally’ (Jaspers, 1997: 301). In Jaspers’ vision of psychiatry, understanding seeks self-evident meaningful connections between psychic events, while explanation seeks rules of cause and effect through inductive generalization.^1^

Jaspers sees ordinary empathy as essential to understanding, and as a process whereby ‘[w]e understand other people, not through considering and analysing their mental life, but by living with them in the contexts of events, actions and personal destinies’ (Jaspers, 1912/1968: 1315). However, he also believes that, alone, this ordinary empathy produces ‘mere sympathetic understanding’, which falls short of ‘explicit knowledge’, and is ‘“merely subjective” in a derogatory sense’ (p. 1315).

Understanding is based on ordinary empathy, but is regimented and supplemented in several ways. The ideal (albeit unreachable; see Gatta, 2014) goal of understanding, for Jaspers, is ‘a fully conscious understanding of mental processes, one that can be presented in definite terms and forms’, by which he means a fully intersubjectively accessible description of conscious experiences (Jaspers, 1912/1968: 1315; see also Wiggins and Schwartz, 1997). With respect to a particular ‘psychic phenomenon’, Jaspers (1912/1968: 1316) thinks that we must start from looking at ‘its genesis, the conditions for its appearance, its configurations, its context and possible concrete contents; also by making using of intuitive comparison and symbolization’. He names three main methods for gathering information: ‘(1) immers[ing] oneself . . . in [the patients’] gestures, behaviour, expressive movements; (2) . . . direct questioning . . . (3) written self-descriptions’ (p. 1316). Jaspers also stipulates that understanding must be *presuppositionless*, an idea I shall discuss below.

Jaspers identifies two kinds of understanding that might play a role in psychiatry. The first kind is ‘static understanding’, which he identifies as the product of ‘phenomenology’ (p. 1322); according to Jaspers, phenomenology consists in ‘representing, defining, and classifying psychic phenomena, pursued as an independent activity’ (p. 1314; cf. Gergel, 2012; Zahavi, 2007).^2^ Static understanding is to be achieved by empathy supplemented by inferential processes relying on ‘indirect hints’ and analogies, as well as questioning patients about their experience, and ideally obtaining written accounts of it (Jaspers, 1912/1968: 1316). The second kind is ‘genetic understanding’:

a unique form of understanding which only applies to psychic events; it grasps as self-evident how one psychic event emerges from another; how a man attacked should be angry, a betrayed lover jealous. (p. 1322)

Both kinds of understanding are, according to Jaspers, achieved by processes which rely upon, but outstrip, ordinary empathy.

Equipped with this distinction between explanation and understanding, Jaspers tries to set up a theoretical foundation for psychiatry. He believes that both explanation and understanding have a role to play in psychiatry. In arguing for a phenomenological psychiatry, he is trying to resist a purely, or even primarily, biological psychiatry. He does so by claiming that some psychiatric disorders and their symptoms can be *understood*, in the technical sense laid out above. He also, however, acknowledges that there is a role for biology, for explanation, in psychiatry; he does this by claiming that there are some disorders/symptoms which cannot be understood – disorders/symptoms which are ununderstandable. Jaspers hands the study and treatment of these disorders/symptoms over to the biological sciences.

The paradigmatic example of an ununderstandable symptom, according to Jaspers, is a delusion – for Jaspers, all delusions are by definition ununderstandable; according to *Diagnostic and Statistical Manual of Mental Disorders*, 5th edition (DSM-5) definition of ‘delusion’, only some delusions count as ununderstandable.^3^ All the biographical interrogation and empathetic intuition in the world will not enable the psychiatrist to work out why, for example, a sufferer of Cotard delusion believes that they are dead, or a sufferer of Capgras delusion believes that their spouse has been replaced by a perfect imposter. As such, Jaspers claims, they fall under the remit of biology, which ought to look for lawlike generalizations to explain the presence of such delusions causally.

## Whence understanding?

### Dilthey and the antipositivists

Perhaps the most influential version of the understanding–explanation distinction is owed to Dilthey, although he builds on the earlier work of Johann Gustav Droysen (Burger, 1978; Maclean, 1982; Udehn, 2002: 26–7). In turn, Droysen’s use of the term ‘*Verstehen*’ appears to be taken from the Christian theological tradition of transformational scripture interpretation (Burger, 1978: 7). Burger takes this to indicate Droysen’s wish to emphasise the greater similarity between their procedure and this transformational-religious procedure than between their procedure and natural science (for helpful discussion on the background of the science–theology rift, see Hobart, 2018).

For the most part, Jaspers’ presentation of the distinction closely follows Dilthey’s. Indeed, even Heidegger praises Jaspers for his generous reception of Dilthey’s work (Scharff, 2018: 56). Dilthey (1974: 15) claims that in studying natural objects, we seek to ‘place objects in the relations of cause and effect’. However, he argues along Humean grounds that all we are really doing is finding regularities in the coexistence and succession of sensory stimuli, since we may only ‘know objects from without through our senses’. We attempt to reconstruct ‘the living relation’ between objects through an intellectual interpretation performed by abstract thought (p. 15). This is characteristic of explanation: attempting to make causal claims about natural objects by interpreting and abstracting from regularities in our perceptions of those objects.

However, we are not limited to perceptions and sensory stimuli in our studies of the subject matter of the social sciences, according to Dilthey. Instead, there is sufficient ‘psychic unity of mankind’ (Truzzi, 1974: 9) that we may, as it were, put ourselves in another’s shoes and understand from within – we may ‘reproduce any other person’s mental life’ (Dilthey, 1974: 12). This process of reproduction relies on intuition, empathy, imagination and a ‘*skilled* reproduction’ of the circumstances and personality of the individual who is to be understood (original emphasis).

Understanding is also discussed by Max Weber, who also influenced Jaspers (Manasse, 1981). The importance accorded to understanding by Weber is best understood as an attempted synthesis of Dilthey’s views, and the view of a rival anti-positivist, Heinrich Rickert. Rickert was a contemporary of Dilthey, and although he was Dilthey’s ally in resisting the positivism of Comte and Mill, he did not believe that Dilthey’s view of the difference between natural and social science was correct. Instead, he claimed that the difference between the social and natural sciences lies in the fact that historical events are unique and unrepeatable, whereas the events studied by natural scientists are repeatable and general (Truzzi, 1974: 18–19).^4^

Dilthey, Rickert and Weber between them offer three accounts of the justification for this methodological difference. These are that:

- the goals of the social sciences are different from those of the natural sciences, aiming at humanistic insights rather than laws or universal abstractions (Truzzi, 1974: 9);
- we can reconstruct the mental life of humans and thereby understand the products of their mental life (which together constitute the subject-matter of the social sciences), but we can only perceive the objects studied by the natural sciences (p. 12);
- the events studied by the social sciences are unrepeatable, whereas the events studied by the natural sciences are repeatable.

None of these justifies the application of the method to psychiatry. For example, it may be that Jaspers thought psychiatry ought also to aim at humanistic insights. However, it seems obvious that this is false – psychiatry ought to aim to help people who suffer psychiatric disorders, not to offer us such insights.

The second justification seems to highlight something interesting: we can empathize with someone with schizophrenia, but we cannot empathize with a sub-atomic particle, or any of the objects referred to in physical theory (at least, not any of those of which I am aware). This suggests that there may be a methodological and epistemological difference between psychiatry and the lower-level sciences: empathy might play a role in psychiatry, but not in those lower-level sciences. However, this does not go anywhere near far enough to justify applying the full-blooded distinction between explanation and understanding.

Finally, there is the claim that the events studied by the social sciences are unrepeatable, whereas the events studied by the natural sciences are repeatable. It is hard to assess this claim without a metaphysics of events. For the sake of argument, I will assume a Davidsonian metaphysics of events, as particulars (Davidson, 1970). On this view, events are either trivially unrepeatable, or the question of their repeatability must always be assessed relative to a description. For example, ‘me sitting down on the sofa’ is repeatable; ‘me sitting down on the sofa at 10:37pm on the 29 October 2019’ is unrepeatable; ‘a coup d’état’ is repeatable *qua* coup d’état (there can be more than one coup, and we can make generalizations about coups); ‘the 1997 Cambodian coup d’état’ is unrepeatable.

Similarly, ‘someone getting depression’ is repeatable in that we can study multiple cases of people getting depression and make generalizations about how it happens; ‘the first time Sam Willis got depression’ is unrepeatable. Likewise, ‘two electrons becoming entangled’ is repeatable, whereas ‘the specific time these two specific electrons became entangled’ is unrepeatable. We can allow for the sake of argument that history ought to study events only under descriptions whereby they are unrepeatable, rather than seeking inductive generalizations. However, this simply does not seem to be the case in psychiatry. While psychiatrists certainly ought to be interested in unrepeatable events like ‘the first time Sam Willis got depression’, they ought also to be interested in repeatable events (or events under repeatable descriptions) like ‘someone getting depression’.

### Husserl

Jaspers breaks from Dilthey’s presentation of the distinction between explanation and understanding in one significant way. From his reading of Husserl, Jaspers takes the idea that understanding should be ‘presuppositionless’ in order to rest on a more secure epistemological foundation. However, he and Husserl do not mean the same thing by ‘presuppositionlessness’. Additionally, they do not have the same goals: even if they meant the same thing by ‘presuppositionlessness’, Husserl’s justification(s) of presuppositionlessness would not generalize to Jaspers’ notion.

Jaspers thinks that understanding, like Husserlian phenomenology, requires ‘the strict exclusion of all assertions that cannot be entirely performed phenomenologically. Every epistemological investigation must be carried out on purely phenomenological grounds’ (Husserl, 1900/1970: 263). However, Jaspers means something entirely different: understanding must be ‘phenomenological’ in being directed to the mental *qua* mental, and ‘presuppositionless’ in being uninfluenced by metaphysical and scientific, especially materialist, theories of the mind. According to Jaspers (1912/1968: 1316), claims about the mechanisms that underlie phenomena and theoretical representations of those phenomena are to be excluded from phenomenology and understanding: ‘we must set aside all outmoded theories, psychological constructions, or materialistic mythologies of cerebral processes’.

Jaspers seems primarily to want understanding to be ‘presuppositionless’ in setting aside metaphysical and scientific theories of mind. In fact, there is a whole class of ‘presuppositions’ that he sees as essential to understanding (because they are essential to the operation of ordinary empathy, in his view). Specifically, biographical information plays a major role in understanding. He claims that ‘[w]e understand other people, not through considering and analysing their mental life, but by living with them in the contexts of events, actions and personal destinies’ (p. 1315). For this reason, Jaspers accords great importance to the biographical interview in the practice of psychiatry (Kolle, 1981; Vlasova, 2017). This is incompatible with Husserl’s notion of presuppositionlessness and with his aims for phenomenology (see Scharff, 2018: chapters 2, 3).

This reflects Jaspers’ disagreement with Husserl over the nature of phenomenology: Jaspers regarded it as a form of descriptive psychology, not the transcendental procedure Husserl takes it for (discussed further below).^5^ He suspected that his view differed from Husserl’s. In Jaspers’ words:

Husserl impressed me . . . although his phenomenological method did not strike me as a philosophical procedure. I took it – as he himself did at first – for descriptive psychology . . . In 1913, when I told him I still failed to understand what phenomenology really was . . . he replied, ‘You are using the method perfectly. Just keep it up. You don’t need to know what it is; that’s indeed a difficult matter.’ (Jaspers, 1969: 6–7)

Additionally, in as much as Jaspers understood Husserl’s version of phenomenology, he had a deep distaste for it. He accused Husserl of ‘hav[ing] committed the most naïve and pretentious betrayal of philosophy’ in his aspirations for a scientific philosophy (p. 7).

This, in turn, reflects a difference between Husserl and Jaspers’ philosophical goals. Husserl’s goal was a scientific philosophy, built on elucidation of the essential structures and principles that determine all possible experience; this consists in part in explicating necessary relations between the intentional contents (*noema*) of intentional, psychic acts. He did not see this as a psychological procedure, at least in his later work, because he understood these necessity relations as having the same ‘objective’ and non-psychological status as the laws of maths and logic, even though they are intuited (see especially Husserl, 1977: 14–17). Rockmore (2011: 131) claims that this move requires ‘a supposed parallel between subjective experience and objectivity’.

Jaspers’ philosophical goals for phenomenology, conversely, were significantly less to do with uncovering a realm of foundational necessary truths. Instead, they were to reveal and lift self-imposed limitations in the way humans live their lives and structure their thinking. His *Psychologie Der Weltanschauungen* (Jaspers, 1919) offers a typology of mental attitudes and psychological configurations which he calls ‘worldviews’; he uses this typology to argue that all such worldviews are partly pathological, incorporating harmful elements ‘into which the human mind withdraws in order to obtain security amongst the frighteningly limitless possibilities of human existence’ (Thornhill, 2008; for criticism, see Heidegger, 2009). Jaspers’ quasi-Husserlian addition of ‘presuppositionlessness’ to Dilthey’s scheme does not therefore point to any justification from Husserl for his use of the distinction or his amendment of that distinction, either in his philosophy or in his psychopathology.

However, Husserl (1977, 1989) does express some limited approval of Dilthey’s distinction. Even here, Husserl’s arguments for the distinction are not amenable to Jaspers’ purposes. Husserl finds three insights in Dilthey’s presentation of the understanding–explanation distinction, two intended by Dilthey, one not intended by him. The first insight is the need for a ‘descriptive psychology drawing purely upon intuition’ (Husserl, 1977: 6). He sees this enterprise as entirely separate from a ‘natural-scientific’, ‘explanatory’ psychology that works by a ‘hypothetico-constructive procedure’, i.e. formulating and testing laws by experiment (p. 9). For Husserl, ‘understanding’ as defined by Dilthey is a flawed attempt to explicate the proper epistemic goal of this descriptive psychology, which in his view is ‘its own species of the highest performance of clarification’ (p. 6). The second insight Husserl attributes to Dilthey is that only *descriptive* psychology, and not *explanatory* psychology, can serve ‘the socio-cultural sciences as a foundation’ (p. 9).

Why can only a descriptive psychology play this role? This is the final insight, the ‘marvellous paradox which Dilthey had not noticed’, that ‘mak[ing] individual mental acts and products intelligible means nothing other than making their individual necessity apparent’ (Husserl, 1977: 12). For Husserl, ‘understanding means nothing else’ than seeing the ‘individual necessity’ of the connections between intentional contents (pp. 12–13). In other words, Husserl’s limited approval of Dilthey’s scheme is entirely dependent on his *transcendental* goals for phenomenology. It therefore does not generalize to Jaspers.

### The mind–body problem

I believe, therefore, that Jaspers has a dual motivation in setting up the framework of understanding and explanation. One is positive – Jaspers is keen to use scientific methods developed in the antipositivist tradition that let us make rigorous use of empathy in psychiatry, making up for a perceived lack in the methods of the natural sciences. One is defensive – to limit the influence of biological and naturalistic approaches in psychiatry. This latter aim is achieved by a methodological stipulation that when examining the mental *qua* mental, one must entirely ignore information acquired with the outlook and methods of the natural science, especially information about underlying mechanisms. Further support for Jaspers having this aim is that he is highly suspicious of the analogies between ‘mental’ and ‘physical’ health, disease, treatment and recovery (Jaspers, 1997; Kolle, 1981).

This can to some extent be seen as Jaspers’ solution to a perceived mind–body problem (see also Bolton, 2004). This also goes some way towards accounting for the continued uptake of Jaspers’ framework: the idea of a mind–body problem is extremely influential in psychiatry. It does not, however, justify its uptake. The idea of a mind–body problem is extremely nebulous (see Berrios, 2018; Pernu, 2017),^6^ arguably illicitly lumping together several importantly distinct issues (see Rorty, 1982). One worry here, expressed by Gough (2021), is that the mind–body problem is an unhelpful idea in psychiatry, and that psychiatry ought to take an attitude of ‘naïve naturalism’ towards the mental (see Hornsby, 1980, 2001; see also: Berrios, 2018; Kendler, 2012). Interestingly, Fuchs (2022: especially p. 8 and footnote 4) intimates a similar position, claiming that the problem of mental causation should be reconsidered as the problem of ‘personal’ causation.

It is easy to see why Jaspers would have believed in the epistemological and methodological independence of the mental. Husserl, Jaspers, Dilthey and Heidegger all expressed a view of natural science and its ‘explanations’ as trafficking in observation and experiment and aiming at the discovery of lawlike regularities (see Scharff, 2018: ch. 2). They saw this as insufficient for studying human beings, for a variety of reasons. All aimed to capture the idea that it missed something important (see especially Heidegger, 2009). Whether or not this view of natural science was justified at the time, it was largely dominant. Despite this, it is far from clear whether it accords or has ever accorded with natural-scientific practice (e.g. Cartwright, 1983, 1999; Feyerabend, 1993, 2001; Mitchell, 2002; Wimsatt, 2007).

This is important because Jaspers is often praised as a forerunner of methodological pluralism in science and psychiatry (e.g. Ghaemi, 2007; Owen, 2007). This might perhaps be developed into a defence of adopting and amending the distinction between understanding and explanation, so I wish to reject the idea here. Pluralism is not just believing in more than one thing; Jaspers is only as much a forerunner of methodological pluralism as Descartes is a forerunner of metaphysical pluralism. Let us take understanding as a scientific method in Jaspers’ scheme. It is then one of precisely two scientific methods in his scheme: explanation, the method of all and only natural sciences, and understanding, the method of all and only human sciences. Human beings are appropriately studied by natural science, in Jaspers’ scheme, exactly in as much as they cannot be studied using understanding, the method of human science.^7^

Jaspers argues for understanding as part of psychiatry only by falsely homogenizing the natural sciences as sharing a single well-defined method (see also Ferry-Danini, 2018, 2019). His view of both the natural sciences and the human sciences is completely antipluralistic, assigning each with exactly one method and one epistemic goal. Believing that science, or the world, divides into two neat, homogeneous and mutually exclusive realms is not a ‘pluralism’ worth the name.

Even setting these issues aside, Jaspers’ stipulation of independence is a deeply awful idea if the mental/personal is *not* in fact methodologically and epistemologically independent of its underlying mechanisms and of information best gleaned through the natural sciences (for further critique, see Bentall, 2003: 25–9; Gough, 2021). There is no such independence, and the stipulation must be rejected (see especially Gough, 2021; Kendler and Campbell, 2014). The key point, made repeatedly in the literature, is that just as empathy can be enhanced and made more rigorous and useable if it is constrained and informed by information about an individual’s life and circumstances, so too can it be made more rigorous and useable if it is continuously informed by and used to inform theories about the underlying mechanisms.

For example, Gough (2021) discusses the influential two-factor account of delusions, which lets us start making sense of monothematic Cotard and Capgras delusions by compiling information from neurology, perceptual psychology and cognitive neuroscience (e.g. Coltheart, 2007; Coltheart, Langdon and McKay, 2011; Turner and Coltheart, 2010). Cotard delusion, on this account, usually begins because the patient’s autonomic nervous system, responsible for affect, fails; the person feels disembodied and numb, and it occurs to them that perhaps they are dead. Capgras delusion usually begins because the patient’s autonomic response to facial recognition is severed; where normally seeing their partner’s face would trigger a complex affective response, it triggers nothing, even though they recognize the face as looking exactly like that of their partner; it occurs to them that perhaps, despite looking like their partner, the person they are facing is not actually their partner – a perfect imposter.

In each case, the thought that occurs to them is primarily produced by informationally encapsulated perceptual systems which cannot holistically assess plausibility; this thought is, by default, accepted as a belief (inputs from the perceptual system are, it is hypothesized, endorsed by default). Normally, however, such a thought would simply be rejected as absurd, in the light of a more general assessment (Turner and Coltheart, 2010). In sufferers of delusions, this is not the case. It is proposed that this is due to some second factor, often damage to an area of the right prefrontal cortex responsible for attention; in other words, the patient does not have enough control of their attention to make the effort to assess and reject the belief once it is, by default, accepted; it is also for this reason that the belief fails to bring about some of its expected behavioural consequences (Davies and Egan, 2013).

Such a story seems to reveal the content of the delusions, and the process by which the delusions are produced, as meaningful; it enables someone who does not suffer from the delusion to empathize with the sufferer – we can make sense of what it is to have a limited attention span, to see something weird, to feel numb, and so on. The accurate description of the patient’s experience, and our ability to grasp how it arose, that is, static and genetic understanding, are both enhanced by drawing on information about underlying mechanisms. Jaspers, however, rules out this possibility *a priori*.

## What remains?

There are several proposals for revising the notion of understanding so that it might continue to play an important role in psychiatry. Some of them attempt to revise the distinction between understanding and explanation so that it marks an important divide within psychiatry – although many such proposals are not explicitly offered as revisionary (see the discussion by Gough, 2021, of Kendler and Campbell, 2014). Since Gough focuses on dismissing these proposals, I will not retread that ground here.

An interesting recent proposal is that of Fuchs (2022). He begins by arguing that genetic understanding can have a legitimate causal-explanatory role thanks to his prosaic view of top-down causation (see his §3), and that static understanding can have a legitimate explanatory role in tracing the development of full-fledged symptoms from ‘basal disturbances’ in the structure of consciousness (pp. 10–11). Fuchs argues for a revisionary view of understanding as a form of explanation. This goes beyond the idea that explanation and understanding can inform one another – to understand, on this view, *just is* to explain in a particular way. But to claim that understanding is a form of explanation is surely to deny the existence of ‘understanding’ and ‘explanation’ in any form that the antipositivists or the phenomenologists would have recognized.

Perhaps Fuchs (2022) means that some version of the *method* that Jaspers calls ‘understanding’ should be part of the toolkit of natural science, that is, a form of ‘explanation’. As I acknowledged in section “Dilthey and the antipositivists”, humans are unusual among objects of inquiry in that we can learn about them through empathy as well as other means, and while this does not justify applying a full-blooded explanation–understanding distinction, it does highlight something epistemically unusual about studying human beings.

This is not how Fuchs understands his proposal. As he puts it, ‘[p]henomenological psychopathology . . . has long since gone beyond the description of psychic experience in the sense of Jaspers’ (p. 10). For Fuchs, understanding ‘goes beyond’ Jasperian understanding ‘by exploring the transcendental basic structures of consciousness such as embodiment, temporality, self-experience and intersubjectivity’ (p. 10). This gives access to a ‘pre-reflexive dimension of experience’, enabling the psychiatrist to ‘extend his [*sic*] understanding to phenomena that might otherwise be regarded only as incomprehensible products of brain dysfunction’ (p. 10). This has a significantly more Husserlian flavour, according well with Husserl’s (1977) description of a phenomenological psychology.

Exactly what, then, does Fuchs defend when he defends a role for understanding in psychiatry – what does he mean by ‘understanding’? Perhaps Fuchs is defending only a loose umbrella concept of understanding, where ‘understanding’ is understood as a generic label for introspection- and empathy-informed methodologies. If this is the correct reading of Fuchs, then Fuchs is largely in agreement with Gough (2021) and Ferry-Danini (2019).

It would not be unusual for Fuchs to treat the term as a mere umbrella term. Parnas and Sass (2008: 262)^8^ defend the importance of Jaspers’ concept of understanding by claiming merely that ‘elements of understanding are necessarily involved in . . . descriptive processes . . . because [these processes] are always influenced by the search for meanings’. These descriptive processes are typification (making explicit and critiquing the concepts and prototypes we use to navigate the world; pp. 258–9), exploration of subjective *structures* (as opposed to exploration of mere *contents* of experience; p. 260), and the search for invariances (necessary features of a kind of experience, discovered by a process of ‘eidetic-imaginative variation’; pp. 259, 262). Fuchs (2008) is keen to add to this list of techniques the nondescriptive process of tracing symptoms and illnesses back to their ‘roots’ in ‘prethematic’, ‘prereflective’ or ‘basal’ experience.

However, he is not treating ‘understanding’ as an umbrella term either. A particularly telling passage is the following:

This concept of understanding . . . does not mean psychological or empathetic understanding. Rather, it is an understanding . . . informed by an explication of the implicit constitutive structures of conscious experience. *Phenomenology in this sense* seeks to find the ‘logos’ of the phenomena in themselves, *not in underlying subpersonal mechanisms*. (p. 280, my emphasis)

This characterization of understanding has four interesting components:

- First, Fuchs-ian understanding is similar to Jasperian understanding in being based on empathy, but with some additional steps that take it beyond ordinary empathy.
- Second, however, unlike Jaspers this extra step consists in a Husserlian explication (based on a phenomenological reduction (Fuchs, 2008); see also Parnas and Sass, 2008: 260) of the underlying structures of consciousness and the laws that hold between purely psychic phenomena – not a careful biographical interview procedure, nor an attempt to open oneself up to the gestures, expressions, words and world of another.
- Third, Fuchs (2008: 280) appears to commit himself to precisely the same problematic principle as Jaspers, that understanding cannot be informed by knowledge of ‘underlying subpersonal mechanisms’. Parnas and Sass (2008: 270) also commit to the ‘autonomy of the phenomenological’.
- Fourth, and strangest of all, Fuchs appears to treat ‘understanding’ and ‘phenomenology’ (perhaps more charitably read as ‘phenomenological psychology’) as *synonyms*.

The third aspect of this characterization renders the notion, in my view, unhelpful for psychiatry: the same goals are better achieved without ruling out swathes of *de facto* helpful information as irrelevant by *a priori* fiat (see section “The mind-body problem”). This third aspect also highlights a problematic aspect of the idea of ‘understanding’ in contemporary psychiatry: it remains closely associated with the idea that the natural sciences (or ‘biomedical approaches’ in a modern idiom) have overreached, and with a call for their retreat from the human domain.

If we drop this principle, however, then all that remains of Jaspers’ and Dilthey’s notions in Fuchs’ (2022) proposal is the idea of a method that uses empathy in some way. It is far from clear that it is useful to label such a method ‘understanding’. This version of ‘understanding’ is no longer the philosophically, epistemologically and methodologically weighty notion introduced by the antipositivists to resist the overreach of (natural) science into the human realm. Like other versions of ‘understanding’, it *does* at least involve a suggestion for making rigorous use of empathy – in particular, by drawing heavily on the resources of phenomenology. However, it is unclear that this procedure is appropriately labelled ‘understanding’, when it is more or less identical with phenomenological psychology, and already called ‘phenomenology’ by Fuchs (2008).

There is a deep duality to the notion of understanding. On the one hand, it serves (in Fuchs as in Droysen) as a call for the sciences to remedy a genuine problem: the historical poverty of vision in the dominant epistemic goals of sciences in the human realm (see also Habermas, 2015; Hesse, 1980), and the widespread neglect of many approaches to the human realm, stemming from a misplaced scientistic squeamishness about making use of information gathered by introspective and empathetic processes. On the other, the solution implied (from Droysen to Fuchs) is one where the natural sciences ‘back off’ from (certain parts of) the human realm, and leave (those parts of) the human realm entirely to a methodology based on philosophy, introspection and empathy, one built on a deep suspicion of experimentalism (consider also Hobart, 2018).

The problem is that properly construed, experimentalism, naturalism and biomedicine need empathy- and introspection-informed approaches, and vice versa. The solution here is not to reject science as we know it in favour of Husserlian phenomenological psychology. Part of the solution is to integrate phenomenology and its findings into science as we know it (as Fuchs, 2022, argues). Another part of the solution is to integrate scientific findings, including findings about underlying mechanisms, into the evidence base of phenomenology (see also Kendler and Campbell, 2014). The aim should not be to hold separate the naturalistic or materialistic aspects of phenomena, explananda and explanations from the mental, personal or humanistic aspects (nor should we organize, e.g. psychiatric interventions in this way, *contra* Pernu, 2022).^9^ Instead, the aim should be to turn best practice in psychiatry into a regulative ideal (see also Kendler, 2012): the seamless integration of *all* relevant kinds of information, unbiased by the frankly parochial scepticism of vast swathes of relevant information often associated with both materialist *and antimaterialist* worldviews.

## Notes

1. Two clarificatory remarks on the nature of these ‘meaningful connections’ are relevant: first, they need not be rational; second, they may well be causal, but instances of singular rather than general or law-governed causation (Hoerl, 2013, 2019).

2. Here, it is especially questionable how closely related Jaspers’ ‘phenomenology’ is to Husserlian phenomenology.

3. American Psychiatric Association (2013: 87).

4. It is from Rickert’s work that the distinction between *idiographic* and *nomothetic* explanation derives (Truzzi, 1974: 18–19).

5. For anyone worried that a non-Husserlian phenomenology is no phenomenology at all, see Rockmore (2011, esp. chapters 2, 3).

6. This is in part because the idea of the ‘mind’ is itself rather nebulous. Consider, for example, the futility of trying to identify the concept of mind with one of Husserl’s concepts of *pure ego*, *psychic ego*, *psyche*, *spirit*, and *soul* (*psyche* is perhaps the most popular option, but consider, for example, Moran, 1996).

7. An idea that, as Bentall (2003) highlights, preempts Dennett’s (1987, 1991) idea that we drop out of the ‘intentional stance’ only when faced with sufficient malfunction.

8. As a historical point, it is worth stressing the importance of Fuchs, Parnas and Sass in leading the contemporary resurgence of phenomenological methods in psychiatry.

9. Nor, in my view, is the solution to dive deep into speculative metaphysics (*contra* O’Leary, 2021) – debating purely theoretical propositions such as the existence of fundamental, irreducible qualia does not help us to take reports of experience seriously. More importantly, O’Leary spends much of the paper showing that the relevant terms are confusing and ill-defined. It is at this point that we part ways. I would prefer to avoid that baggage, insistently adopting a naïve naturalism that refuses to accept the initial conceptual division of the world into mind and body. O’Leary does not successfully circumvent the issue in my view. Instead, she defines all her terms by a few spurious appeals to a mythical philosophical consensus, passing off stipulative definitions as simply obviously correct. There is no such consensus, least of all on the meaning of ‘dualism’ and ‘physicalism’. For example, to someone with my philosophical background, hers looks like a deeply contentious and uncharitable account of nonreductive physicalism.
